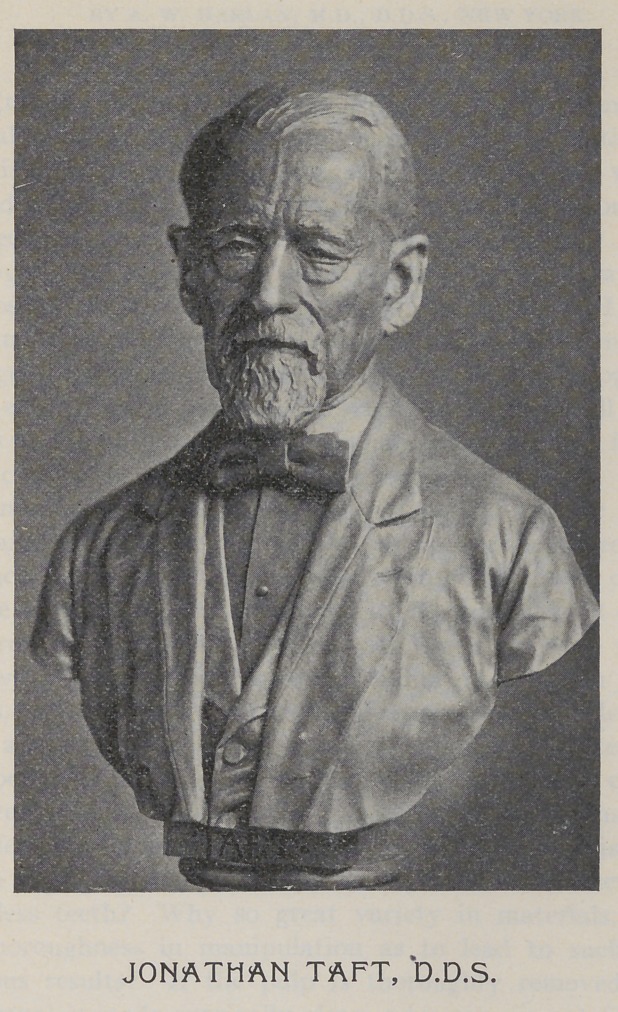# Event and Comment

**Published:** 1905-04-15

**Authors:** 


THE
### DENTAL REGISTER.

Vol. LIX. April 15, 1905. No. 4.

### EVENT AND COMMENT.

### Bust of Dr. Taft.

We present in this issue a half-tone picture of a bronze bust of Dr. JONATHAN TAFT, which was presented to the University by the alumni of the College of Dental Surgery of the University of Michigan, February 17th. The bust is the work of Dr. Victor H. Jackson, of New York city, the noted orthodontist and author of the text-book on this subject. Dr. Jackson is a graduate of the class of 1877, and was a very great admirer of Dr. Taft, and a warm personal friend. The work has consumed the spare time of Dr. Jackson for several years, and is a remarkable piece of work from the hands of a man who is only an amateur at the business and not a professional. We are sure our readers will concur in the statement that Dr. Jackson has caught the characteristics of Dr. Taft's expression in a remarkably perfect manner. The bust will be placed in the art gallery of the University until the new dental building is erected, when it will be suitably placed in that building. The bust is so much more satisfactory than an oil painting and is also much more durable, and it is a grand idea to have so life-like a picture to refresh the memories of those who knew and loved Dr. Taft. It would seem that it would be very appropriate if a copy of the bust could be placed in the Ohio College of Dental Surgery also, as it was here that he began his career as a teacher and to this school he gave a large part of the most active years of his life.

**Metal versus Vulcanite Plates.**

The comparative merits of metal and vulcanite dentures has for many years been determined by conditions entirely unworthy of the progress or development of oral sanitary science. The verdict has gone to vulcanite more often than it should for the financial reason alone, and yet we feel that had the rank and file of the profession been equipped to stand for the best, that metal plates should have made a better showing. Metal plates cost more money; they are more difficult to construct properly; hence people have not been willing to invest in them and the profession has not urged them, although there has never been a time when a rubber plate has been considered as good from either the utilitarian or hygienic standpoint. The great development of skilled metal workers in the profession in recent years, because of the demand for crown and bridge-work, has had its influence in turning this ability to other features of the prosthetic field. The importance of cleanliness in connection with the various forms of bridge-work has also turned the attention of many to notice that the ordinary vulcanite plate is not an ideally clean affair after it has been worn for a year or two. The inexpensiveness of aluminum and the improved appliances for manipulating it has caused it to supplant rubber as a base for full dentures in most well-conducted offices. The shortcomings of aluminum have made the step easy to gold plates, and there seems to be a growing tendency to the use of this metal as a base for both partial and full dentures. It is a good sign, for, in our judgment, no plate is superior to a well-made and properly-finished gold plate, for either partial or full dentures. With the improved swaging appliances, it is not much more difficult to make than an aluminum plate and outside of cost of material should not exceed the ability of many to pay.

**Extracting Teeth for Regulating.**

The Second District Society of New York had this subject up for discussion at its February meeting. Judging from the verbal reports of the meeting that have come to us we should judge that the contention was well supported by the old school practitioners and just as severely condemned by the so-called new school. This subject is an old one and will never be settled by argument, although the argument is necessary to lead up to the proper basis for its final settlement. As the matter stands now it is the case of practicable against the idealistic. The man who cares most to secure reasonably practicable results without the expenditure of much time or skill will see many advantages in extracting a tooth occasionally to facilitate his operation. While on the other hand the man who makes the highest type to be found in nature his ideal as well as his objective, will never consent to the extraction of a tooth which he knows will preclude the attainment of his ideal. The practical man in all walks of life is glad to have it known that he will compromise his principles rather than not succeed; he considers this a good business method. The same idea to a certain extent prevails professionally. Some men will not sacrifice their ideals in any case, not even to be practicable. Others are willing to take a half loaf if they can get no more. There is no question in our own mind but that the orthodontist who insists on perfect occlusion of the teeth is right, and where this work is done at the proper age there should *rarely* be tolerated an extraction. At the same time we can readily see how men of the most honorable intentions can't see any harm in extracting when the results attained seem to justify the loss of a single tooth.

**Length of the College Course.**

AFTER all the discussion and effort of the past two years, it seems that something definite will be the outcome in the establishment of the length of time necessary for a student to qualify under the rules of the National Board of Examiners. The committee on colleges of this body has issued a statement which will constitute its standard of reputability and arranged it with an option that will accommodate both the long and short term advocates. The following statement will contain the elements of their proposition.

"For matriculation or registration, graduation from an accredited high-school or its full equivalent, examinations of credentials and equivalents to be placed in the hands of an acceptable appointee of the State Superintendent of Public Instruction, where not otherwise provided for by law. Said entrance requirement to be inaugurated not later than the session of 1906 and 1907. The course of instruction shall extend either over a period of four years with four sessions of seven months terms in each session, or over a period of three years with three sessions of nine months in each. The length of term to be adopted with the session beginning in the fall of 1905." The three Chicago schools have announced their intention to begin the three-year course of thirthy-two weeks this fall and adopt the high-school requirement in the fall of 1906. In this case the intention is that the thirty-two weeks shall exclude all vacations. The committee of the examiners' association has accepted this interpretation of their standard as equivalent and will recognize these schools. With these schools and the schools which already maintain their standard, a considerable number of the colleges are already pledged to support the National Examiners' standard, and it looks as though the end of this struggle was in sight.

**Enthusiasm.**

We once heard a lecture by a famous President of the United States on "The Power of Sentiment," which made a great impression on our mind and has caused us to treat sentiment with more respect ever since. We have recently had the good fortune to come in contact with several practitioners of dentistry who have impressed us with the power and value of enthusiasm. We do not, of course, refer to any fanatical excesses over some mythical ideal, but to a wholesome and ardent zeal concerning a special branch of practice. The manifestations of these particular cases are not blatant and offensive, but of that natural and ingenious character that commands respect of all well-meaning people, and that become, as it were, infectious, stimulating all who come within their influence to think and to do. In a community that we know, there came a young dentist and took up a specialty, and he practiced it so well that he, by the power of his example of what could be done by one who starts right and works with energy and enthusiasm, has been almost directly the cause of inducing at least a half dozen other practitioners in the same city to take up various special lines of practice which they were pursuing with similar energy and consequent success. The peculiar and commendable feature of this instance is that there is no symptom of jealousy apparent, but they are most friendly and are together like brothers on every possible occasion. We have watched this little coterie as it has grown gradually and it seems now to have reached the point where it is destined to influence a much wider circle than the profession of its own city or State. We can not help contrasting with it another city not a hundred miles distant, where the conditions are of an entirely opposite type. In this one, each man seems to be self-contained. If he is thinking or doing more than to cobble a few teeth, no one besides himself is wiser, for he never gives out any indication that he gets any more pleasure out of his calling than the food and shelter it provides for him. They have a dental society in that city, which meets occasionally, but no one ever heard of its contributing anything of great value or consequence to the general fund. The fact is there is great need that some one in that place shall get religion—we mean applied religion, so to speak. If some man full of the spirit should settle in that town and set fire to the accumulated junk, it would be the greatest blessing that could come to the people of that city, who are dependent for up-to-date service on a dead profession. There is no reason why a dentist might not get enthusiastic to-day over a dozen different lines of practice. Every one should get busy with one at least.

Dr. S. H. Guilford, in the March *Stomatologist,* makes two objections to the Smith method of prophylaxis. The first is that patients will not consent to the treatment ten times a year; and the second that should they do so the enamel of the teeth would be rubbed so thin as to threaten the value of this tissue to the tooth. The best answer to the first objection that can be made is that experience has demonstrated that patients not only comply with this requirement but do it most willingly. If this is not sufficient let any dentist who doubts submit himself to the operation and be convinced. In regard to the second objection, it is to be supposed that any one who is conscientious enough to take up this line of work will be sufficiently intelligent to know when he is injuring the teeth, and will so modify his treatment as to get only the desired cleanliness without excessive abrasion. We have experienced the treatment from both aspects and know that it is one of the best of the newer practices.

## Figures and Tables

**Figure f1:**